# Proof of concept for the use of trained sniffer dogs to detect osteosarcoma

**DOI:** 10.1038/s41598-022-11013-1

**Published:** 2022-04-28

**Authors:** Agustín Ortal, Aida Rodríguez, María Pilar Solis-Hernández, Miguel de Prado, Verónica Rey, Juan Tornín, Óscar Estupiñán, Borja Gallego, Dzohara Murillo, Carmen Huergo, Juan Luis García-Llano, Serafín Costilla, René Rodríguez

**Affiliations:** 1 Canvida Detection Organization, CP 33212 Gijon, Spain; 2 Instituto de Investigación Sanitaria del Principado de Asturias (ISPA), Hospital Universitario Central de Asturias, Av. de Roma s/n, 33011 Oviedo, Spain; 3 Department of Medical Oncology, Hospital Universitario Central de Asturias, Oviedo, Spain; 4 Instituto Universitario de Oncología del Principado de Asturias, 33011 Oviedo, Spain; 5 CIBER en Oncología (CIBERONC), 28029 Madrid, Spain; 6 Department of Radiology, Hospital Universitario Central de Asturias, Oviedo, Spain

**Keywords:** Bone cancer, Animal behaviour

## Abstract

Sarcomas are mesenchymal cancers which often show an aggressive behavior and patient survival largely depends on an early detection. In last years, much attention has been given to the fact that cancer patients release specific odorous volatile organic compounds (VOCs) that can be efficiently detected by properly trained sniffer dogs. Here, we have evaluated for the first time the ability of sniffer dogs (n = 2) to detect osteosarcoma cell cultures and patient samples. One of the two dogs was successfully trained to discriminate osteosarcoma patient-derived primary cells from mesenchymal stem/stromal cells (MSCs) obtained from healthy individuals. After the training phase, the dog was able to detect osteosarcoma specific odor cues in a different panel of 6 osteosarcoma cell lines with sensitivity and specificity rates between 95 and 100%. Moreover, the same VOCs were also detected by the sniffer dog in saliva samples from osteosarcoma patients (n = 2) and discriminated from samples from healthy individuals with a similar efficacy. Altogether, these results indicate that there are common odor profiles shared by cultures of osteosarcoma cells and body fluid samples from patients and provide a first proof of concept about the potential of canine odor detection as a non-invasive screening method to detect osteosarcomas.

## Introduction

Osteosarcoma is the most common type of primary solid tumor arising from bone tissue^1^. Although it has a relatively low overall incidence (0.3 per 100.000 per year), this type of tumors represent approximately 15% of pediatric tumors^1,2^. As other types of sarcoma, osteosarcomas arise upon the malignant transformation of mesenchymal stem/stromal cells (MSCs) or their derived cell types along the osteoblastic lineage^3–5^. Conventional osteosarcoma, the most common subtype, is always high-grade and is frequently metastatic at the time of diagnosis^6^. Current standard of care, based on an accurate surgery accompanied by chemotherapy, remains largely unaltered for decades and patients with metastatic disease still face dismal 5-year overall survival rates below 20%^2,6^. Therefore, as in other tumor types, an early diagnosis is key to improve the prognosis of osteosarcoma patients. In this regard, already established populations screening methods, such as those available for the early detection of breast, colon or prostate cancer patients, have been successful in improving patients’ survival^7–9^. Nevertheless, these screening programs involve invasive and/or costly methodologies and they are not available for most tumor types, including osteosarcomas.

In order to develop novel non-invasive detection techniques, much attention is being given to the fact that individuals with cancer may release specific volatile organic compounds (VOCs)^10^. These odorous chemicals with low molecular weight can be detected, both, in body-derived non-tumor samples (blood, urine, stool, exhaled breath, etc.) and tumor samples^10^. This cancer-associated “volatilome” profile is the result of specific metabolic changes induced by tumor cells and its detection may provide a fully noninvasive diagnostic and/or prognostic biomarker^11^.

Identification of VOCs in a gaseous mixture can be done by chemical analytical techniques such as gas chromatography linked to mass spectrometry (GC/MS) or by using sensor arrays or “electronic nose” (eNose) to create specific smellprints or VOC profiles^10,12,13^. In addition to these laboratory technologies, the complex olfactory system of dogs has proven its ability to detect VOCs in concentrations of parts per trillion. Indeed, for many compounds, dogs have shown a limit of detection which is lower than the most sensitive mass spectrometry or eNose systems^13^. Thus, apart from being long used for many civilian, military and forensic applications, trained sniffer dogs have also demonstrated their ability to discriminate cancer-associated VOCs in body fluids and tumor samples from patients with non-small cell lung cancer, breast cancer, prostate cancer, colorectal carcinoma, melanoma, or ovarian cancer^14,15^. In order to achieve the most reliable results, the implementation of standardized methods for sample handling and dog training are essential^16^. In this regard, cell lines may provide a convenient source of specimens presenting low sample-to-sample variability and absence of patient-specific confounding odors (stress hormones, medications, etc.). Therefore, the use of cell lines may facilitate training and pilot testing experiments to validate initial hypotheses regarding the suitability of canine scent to discriminate cancer patients^17^.

The ability of sniffer dogs to detect sarcomas has not been previously studied. The objective of this study was to provide a first proof of concept about the potential of using sniffer dogs as a screening method to detect osteosarcomas. To this end, we trained dogs to discriminate osteosarcoma cell cultures from healthy MSCs cultures and then analyzed their ability to detect specific odor signals in new osteosarcoma samples (cell lines and saliva from patients).

## Materials and methods

### Cell cultures, saliva samples and ethics statement

A panel of primary, immortalized and cancer cell lines was used in training and/or testing experiments. The main features of these cell cultures are listed in Table 1. This panel includes two primary cell lines (OST-3 and OST-4) generated from osteosarcoma samples surgically resected at the Hospital Universitario Central de Asturias (Oviedo, Spain) as previously described^18–20^. OST-3 derives from a conventional osteoblastic osteosarcoma resected from a 10-year-old female patient and OST-4 corresponds to a dedifferentiated osteosarcoma from a 69-year-old female patient. The OST-3 and OST-4 cells used in this study do not accumulate more than 20 passages in in vitro culture. In addition, other 5 established osteosarcoma cell lines (143B, Saos-2, U2OS, G292 and MG63) originally obtained from the American Type Culture Collection were used in testing experiments. Since MSCs are the cell type of origin of most sarcoma subtypes, we used two cultures of human bone marrow-derived MSCs (BM-MSCs) derived from healthy donors as non-tumor controls. These control BM-MSCs were respectively a primary culture (BM-45) (Inbiobank, San Sebastian, Spain) and cell line immortalized through the overexpression of hTERT and the inactivation of p53 with the E6 antigen of the human papillomavirus 16 (MSC-2H6)^21,22^. All cell lines were tested to discard mycoplasma contamination using the Biotools Mycoplasma Gel Detection kit (B&M LABS, Spain). To collect samples, cells were seeded in 75 cm^2^ flasks (Corning, Glendale, AZ) and cultured in DMEM (Thermo Fisher Scientific, Waltham, MA) with 10% FBS (Biowest, Riverside, MO), 1% Glutamax (Gibco, Thermo Fisher Scientific), and 100 U/ml penicillin/streptomycin (Gibco, Thermo Fisher Scientific) at 37 °C and 5% CO2. Once cultures reached 80% confluence, 4 ml of medium was collected in clear glass vials with screw caps (Supelco, Bellefonte, PA, USA) which were stored at − 80 °C until they were used in odor detection experiments.

**Table 1:** Sarcoma samples and controls used in training and testing experiments.

Cell lines							
	Name	Culture/sample type	Diagnostic/cell type	Grade	Age	Sex	Training/testing
Tumor samples	OST-3	Primary culture	Conventional Osteoblastic osteosarcoma	3	10	F	Training Testing
	OST-4	Primary culture	Dedifferentiated Osteosarcoma	2	69	F	Testing
	143B	Cell line	Osteosarcoma^1^	n.a.	13	F	Testing
	Saos-2	Cell line	Osteosarcoma^1^	n.a.	11	F	Testing
	U2OS	Cell line	Osteosarcoma^1^	n.a.	15	F	Testing
	G292	Cell line	Osteosarcoma^1^	n.a.	9	F	Testing
	MG63	Cell line	Osteosarcoma^1^	n.a.	14	M	Testing
Control	MSC-2H6	Primary culture—immortalized	BM-MSCs	–	34	M	Training Testing
	BM-45	Primary culture	BM-MSCs	–	29	M	Testing
**Saliva samples**							
Tumor	PT-OS#1	Saliva	Conventional fibroblastic osteosarcoma	3	58	M	Testing
	PT-OS#2	Saliva	Conventional Osteoblastic osteosarcoma	3	58	M	Testing
Control	CTL#1	Saliva	Healthy	–	35	M	Testing
	CTL#2	Saliva	Healthy	–	29	F	Testing
	CTL#3	Saliva	Healthy	–	25	M	Testing
	CTL#4	Saliva	Healthy	–	18	M	Testing
	CTL#5	Saliva	Healthy	–	17	M	Testing
	CTL#6	Saliva	Healthy	–	18	M	Testing
	CTL#7	Saliva	Healthy	–	21	M	Testing
	CTL#8	Saliva	Healthy	–	19	M	Testing
	CTL#9	Saliva	Healthy	–	18	M	Testing
	CTL#10	Saliva	Healthy	–	18	M	Testing
	CTL#11	Saliva	Healthy	–	20	M	Testing
	CTL#12	Saliva	Healthy	–	29	F	Testing

*OS* osteosarcoma, *n.a.* not available.

^1^No subtype information available.

Saliva samples were collected from patients diagnosed with osteosarcoma at the Hospital Universitario Central de Asturias. All patients cited in the Hospital’s medical oncology service as of July 2021 were invited to participate. Due to the low incidence of osteosarcomas, only two patients could be enrolled for this pilot study. Negative control samples were also obtained from healthy donors with no history of oncological diseases (Table 1). Part of each sample was diluted 1:10 in the same culture medium used to grow cell lines. Both diluted and non-diluted saliva samples were aliquoted and stored in clear glass vials at − 80 °C. A serial number was written on each sample at the time of collection to identify individual information.

**Figure 1:**
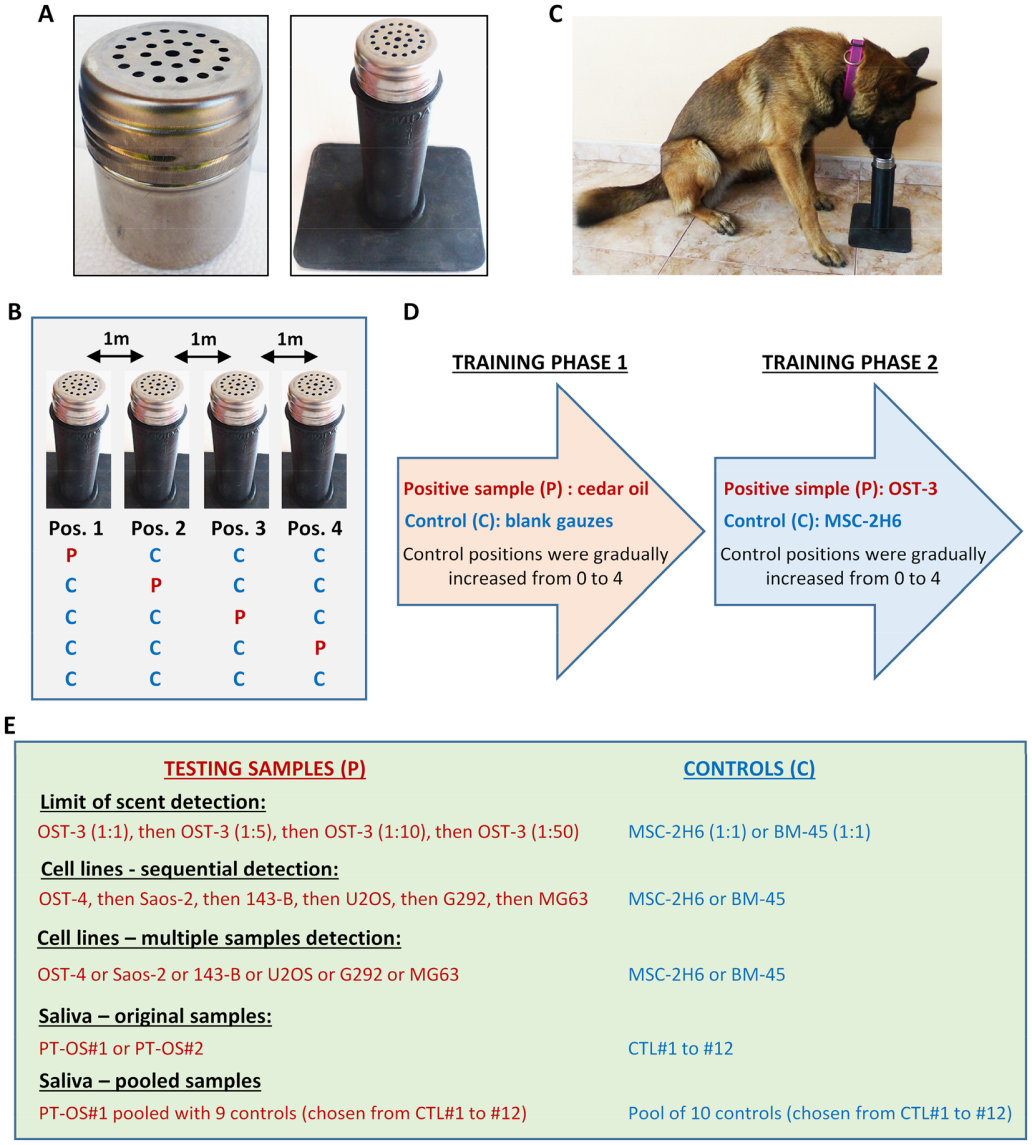
Experimental design and samples used in training and testing experiments. (**A**) Samples were placed in containers with perforated lids (left) which were in turn inserted into cylindrical buckets (right). (**B**) Four sample containers were arranged in a row at 1-m intervals. The dogs were off-leash during the searching experiments and they sniff the positions in sequential order from position 1 to 4. Possible combinations of positive samples and controls are shown. Samples used in each trial, as well as their position, were randomly selected by the assistant. (**C**) Dogs were trained for a “sit stare” final response when finding a target sample. (**D**) Scheme showing the phases of the training and the samples used in each one. (**E**) Positive and control samples used in the different testing experiments.

For training and testing experiments, aliquots of cell culture medium or saliva samples were thawed at 4 °C. Then, a sterile gauze was soaked with 0.1 ml of sample and placed in sample containers with perforated lids which, in turn, were inserted into cylindrical buckets so that the perforated lids protruded and exposed the odor of the sample (Fig. 1A). Thawed aliquots were kept at 4 °C and used for up to week.

Patient samples were obtained at the University Central Hospital of Asturias. All experimental protocols have been performed in accordance with institutional review board guidelines and with the Declaration of Helsinki and were approved by the Institutional Ethics Committee of the Principado de Asturias (reference CEImPA 2021.340). Informed consent was obtained from all participants.

Training and testing protocols with sniffer dogs were carried out in accordance with the institutional guidelines of the University of Oviedo and the Spanish legislation. Ethical review and approval was not required for the animal study because it involved client-owned animals with the best practice veterinary care and these animals were not subjected to painful or distressful protocols. Written informed consent was obtained from the owners for the participation of their animals in this study.

### Dogs and experimental setup

Selection of dogs was based on the following inclusion criteria: (i) dogs had to be clinically healthy, (ii) they had to be regularly available for training and (iii) they had to be previously trained for scent-based searches. Regarding this last requisite, we have not found dogs previously trained for cancer detection in Asturias (a region of 1 million inhabitants), therefore, we decided to enroll dogs that have been trained and used in the search for missing persons, since they are familiar with training procedures. Therefore, two female Belgian Malinois dogs previously trained for the search and rescue of missing people were used in this study. They were a 1-year-old daughter (dog#1, Nai) and her 7-year-old mother (dog#2, Moon).

Training and testing experiments lasted for 16 months with several rest intervals of a maximum of 3 weeks when deemed necessary by the trainer. During these periods, the dogs were kept under appropriate conditions with veterinarian surveillance, as required.

In training and testing experiments up to four identical buckets containing sample containers were arranged in a row at one-meter intervals as previously described^15,23^ (Fig. 1B). The dogs were free and not guided by the trainer during the search for the target specimens and each round of searching started with the trainer command “search”. They were trained for a “sit stare” final response when finding a positive target sample (Fig. 1C). The criteria to define positive and negative detections was similar to that of previous studies^24^. Thus, a correct detection was defined as: (i) identification of the target specimen by sitting in front of the bucket that contained the positive sample and maintain this position for more than 2 s, which are considered as True Positive (TP) identifications, or (ii) sniffing while ignoring control specimens, which are considered True Negative (TN) identifications. An incorrect detection was defined as: (i) identification of the control specimen as the target specimen, considered as False Positive (FP) identifications, and (ii) sniffing without sitting in front of the target specimen, identified as False Negative (FN) identifications. Hesitations longer that 2 s before giving a response were also considered as FN or FP identifications depending on whether the sample was a positive sample or a control. As guiding principle for training, a correct detection was marked with a clicker and rewarded with food.

We defined a trial each time the dog moves along the line of buckets until it marks a positive. A session was defined as all of the consecutive trials completed by the dog. Training and testing sessions lasted between 30 and 40 min, contained between six and fourteen trials and did not occur more than twice a day, with at least 2 h between sessions. A video record was taken for most testing trials along with a written record of the dogs’ behavior at each position. Nitrile gloves were used when handling samples and buckets. Sample containers and buckets were cleaned at the end of each session with 70% ethanol.

### Training

The training of dogs to detect specific odor cues were structured in two phases (Fig. 1D). In the first phase, the dogs were trained to detect the smell of a reference substance not present in the human body. Thus, we use cedarwood oil to train dogs to search for specific odors without confounding their future searches for human-related scents. The dogs were initially rewarded for approaching, smelling and sitting with their nose close to a bucket containing the reference sample. Then, besides the positive sample, we sequentially added buckets containing control gauzes not soaked with any odorous substance until complete all four positions. When the dogs acquired the ability to mark positive samples while ignoring controls and all-blank trials, we move to the following stage. The aim of the second phase was to train dogs to detect specific odors associated to osteosarcoma cells. With this objective, we used cultures of the patient-derived OST-3 primary line. Following the same scheme used in the first phase, dogs were initially trained to detect a bucket containing a gauze soaked in OST-3 culture medium. Then, we sequentially introduced buckets containing culture medium of the control BM-MSC line MSC-2H6 and finally, we trained the dogs to confront all-controls trials. For a dog to proceed to the testing phase, it had to able to discriminate positive samples with sensitivity and specificity rates greater than 80%. In addition, dogs had to show a regular behavior in training trails.

### Testing

Testing experiments were performed with four testing buckets placed in a row and containing either: (i) one cancer sample (from cell lines or saliva) and three controls or (ii) four control samples (Fig. 1B). We performed both non-blind and blind experiments, according whether the trainer were informed or not about the disposition of the samples. Results were recorded by an assistant located outside the testing room in a position where he could view the dog but the dog could not see him. The assistant informed the trainer about the results to allow an immediate reward for correct detections. Different testing experiments were performed using culture media of primary (OST4) and established osteosarcoma cell lines (Saos-2, 143B, U2OS, G292 and MG63) not used in training experiments (Fig. 1E). In a first set of experiments aimed to test the ability of the sniffer dog to detect the different cell lines, it has been exposed to media from the different osteosarcoma cell lines in a sequential fashion, i.e. we performed testing sessions with media from the first cell line, before starting sessions using the second and so on. Then, we performed a set of trials in which the positive sample was randomly chosen by the assistant from the media of all cell lines. To gain insight about the limit of detection of tumor samples of the canine olfactory system, we also confronted the sniffer dog to media from OST3 cultures diluted 1:5, 1:10 and 1:50 times in fresh medium. As non-tumor controls in experiments using cell lines, we used the culture media from both, the cell line used in training experiments (MSC-2H6) and a new BM-MSC line (BM-45). After finishing testing experiments with cell lines, we also aimed to test whether sniffer dogs were able to detect specific odor cues in patient specimens. As a pilot study, we carried out testing experiments using saliva samples from two osteosarcoma patients as positive target and twelve saliva samples from healthy donors as negative controls. As the training were done with culture media samples, we used the saliva samples diluted 1:10 in the same cell culture medium to discard any positive or negative effect of culture medium in dog responses. Finally, to further explore the ability of sniffer dogs to detect osteosarcoma saliva samples we designed testing experiments where Dog#1 was exposed to pools of samples containing an osteosarcoma sample mixed with 9 negative controls in similar proportions (positive pools) and/or pools of 10 negative samples (control pools) (Fig. 1E).

Control samples used in each trial, as well as their position, were randomly selected by the assistant. In all cases at least 6 sessions and more than 60 trials were performed.

### Statistical analysis

Sensitivity and Specificity was used to measure the success of dogs to detect positive samples^15,25^. Sensitivity was calculated according to the formula: TP/(TP + FN) × 100, where TP and FN are the number of true positive and false negative detections respectively. Specificity was calculated according to the formula: TN/(TN + FP) × 100, where TN and FP are the number of true negative and false positive detections respectively. 95% confidence intervals (CI) were calculated using the sensitivity and specificity values of the different sessions. Multiple comparisons of the data were performed using the one-way ANOVA, Tukey’s test. p < 0.05 values were considered statistically significant.

## Results

### Training

Both dogs were easily trained to detect the reference substance without hesitations. Thus, both dogs completed the first phase of the training with correct detection rates greater than 99%. During the second phase of training, Dog#1 progressed rapidly and showed a great ability to discriminate the culture media of the primary osteosarcoma cell line OST3. Although Dog#2 also demonstrated its potential to detect tumor samples, it sometimes entered stages of confused behavior with frequent failures. To register the level of detection ability reached at the end of this phase we performed blind experiments using the same sarcoma (OST3) line employed during the training and two different control BM-MSC cultures (MSC-2H6 and BM-45). Dog#1 was able to detect positive samples and discard negative samples with a sensitivity of 97.65% and a specificity of 98.57%, while Dog#2 was slightly less efficient and discriminated tumor samples with a sensitivity of 90.90% and a specificity of 84.78% (Table 2). Despite, having achieved high levels of detection, we decided to discard Dog#2 from further experiments due its irregular behavior. For instance, this dog found difficulties in pay attention to the sample container placed in the first position of the row or when positive samples are repeatedly placed in the same position in consecutive trials. Therefore, we accomplished testing experiments only with Dog#1.

**Table 2:** Scent detection of OST3 cells.

		Dog#1	Dog#2
Blind sessions	Sessions	11	3
	Trials	108	24
	TP	83	20
	TN	207	39
	FP	3	7
	FN	2	2
	% Sensitivity (95% CI*)	97.65 (94.35–100)	90.90 (71.81–100)
	% Specificity (95% CI)	98.57 (97.26–100)	84.78 (70.03–99.97)

*Confidence interval calculated with the values obtained in the different sessions.

### Estimation of the limit of scent detection in osteosarcoma samples

Dog#1 was able discriminate tumor samples diluted 1:5 and 1:10 times from undiluted control with an efficacy similar to that shown with undiluted samples, both in non-blind and blind experiments. However, when 1:50 diluted samples were tested, the dog decreased significantly its ability to correctly detect tumor samples and therefore, the sensitivity dropped to 50% (Table 3, Fig. S1A). These results showed that culture media from tumor cells contained specific odor signatures at a concentration that was at least one order of magnitude above the limit of detection of the canine olfactory system.

**Table 3:** Detection of OST3 diluted samples by Dog#1.

		Not diluted	1:5	1:10	1:50
All sessions	Sessions	21	10	10	1
	Trials	182	75	78	7
	TP	139	56	57	3
	TN	339	146	165	13
	FP	4	2	1	1
	FN	6	2	3	3
	% Sensitivity (95% CI*)	95.29 (91.87–98.60)	96.55 (92.85–100)	95.00 (90.01–99.99)	50.00
	% Specificity (95% CI)	98.76 (97.66–99.96)	98.65 (97.31–100)	99.40 (97.72–100)	92.86
Blind sessions	Sessions	11	4	7	–
	Trials	108	31	48	–
	TP	83	23	37	–
	TN	207	67	101	–
	FP	3	0	1	–
	FN	2	1	2	–
	% Sensitivity (95% CI)	97.65 (94.35–100)	95.83 (87.67–100)	94.87 (89.21–100)	–
	% Specificity (95% CI)	98.57 (97.26–100)	100 (100–100)	99.02 (96.75–100)	–

*Confidence interval calculated with the values obtained in the different sessions.

### Detection of common odor signatures in osteosarcoma cell lines

Next, we exposed Dog#1 to culture media from a panel of primary and established cell lines not previously used during the training. In sequential testing sessions, Dog#1 was able to discriminate samples of OST4, Saos-2, 143B, U2OS, G292 and MG63 osteosarcoma cells from control samples with sensitivity and specificity rates between 95 and 100% in all cases, both in non-blind and blind experiments (Table 4). In these experiments, we did not find statistically significant differences in the ability of the dog to detect the different cell lines assayed (Fig. S1B). Relevantly, Dog#1 had a correct identification of all osteosarcoma cell lines in the first test it was exposed to the samples. Likewise, the dog correctly discarded the control MSC cultures used in these experiments (Table 5). The sequential representation of the sensitivity and specificity rates obtained in consecutive sessions showed that these values were higher than 95% from the initial session for all cell lines (with the only exception of the sensitivity for OST-4, 89%) and remained essentially constant during all sessions performed (Fig. S2).

**Table 4:** Detection of primary and established osteosarcoma cell lines by Dog#1.

		OST4	Saos-2	143B	U2OS	G292	MG63	Total
All sessions	Sessions	6	19	6	6	8	15	60
	Trials	64	196	75	77	89	160	661
	TP	48	107	41	41	62	106	405
	TN	124	454	173	183	212	490	1636
	FP	3	7	3	2	1	4	20
	FN	1	5	0	0	0	0	6
	% Sensitivity (95% CI*)	97.96 (94.52–100)	95.54 (92.92–100)	100 (100–100)	100 (100–100)	100 (100–100)	100 (100–100)	98.54 (97.53–100)
	% Specificity (95% CI)	97.64 (95.85–99.77)	98.48 (97.25–99.82)	98.30 (96.00–100)	98.92 (97.31–100)	99.5 (97.36–100)	99.19 (98.40–99.92)	98.79 (98.28–99.38)
Blind sessions	Sessions	2	1	–	1	–	2	6
	Trials	22	7	–	9	–	29	67
	TP	17	4	–	9	–	12	42
	TN	43	16	–	7	–	98	164
	FP	1	0	–	0	–	1	2
	FN	0	0	–	0	–	0	0
	% Sensitivity (95% CI)	100 (–)	100 (–)	–	100 (–)	–	100 (100–100)	100 (100–100)
	% Specificity (95% CI)	97.73 (93.83–100)	100 (–)	–	100 (–)	–	98.99 (97.21–100)	98.79 (97.61–100)

*Confidence interval calculated with the values obtained in the different sessions.

**Table 5:** Indication (+: correct; −: false) of Dog#1 at first contact with samples used in testing experiments.

Tumor samples									Control samples												
OST4	Saos-2	143B	U2OS	G292	MG63	PT-OS#1	PT-OS#2	MSC-2H6	BM-45	CTL#1	CTL#2	CTL#3	CTL#4	CTL#5	CTL#6	CTL#7	CTL#8	CTL#9	CTL#10	CTL#11	CTL#12
+	+	+	+	+	+	+	+	+	+	+	+	+	+	+	+	+	+	+	+	+	+

Afterwards, we performed blind testing experiments using samples from all the osteosarcoma lines chosen at random in each trial. Similar to the results obtained in sequential detection experiments, Dog#1 detected randomly chosen osteosarcoma cell lines with sensitivity and specificity rates of 96 and 98% respectively (Table 6). These results strongly suggest that osteosarcoma cell lines share common odor signatures that can be detected by a trained sniffer dog.

**Table 6:** Detection of multiple osteosarcoma cell lines by Dog#1.

Blind sessions	Sessions	6
	Trials	97
	TP	71
	TN	283
	FP	5
	FN	3
	% Sensitivity (95% CI*)	95.95 (90.35–100)
	% Specificity (95% CI)	98.30 (94.92–100)

*Confidence interval calculated with the values obtained in the different sessions.

### Detection of saliva samples from osteosarcoma patients

Finally, we aimed to test whether the common olfactory signature detected in cell lines can also be detected in patient specimens. In a pilot study, we found that the dog was able to discriminate saliva samples from two osteosarcoma patients from healthy controls with sensitivity and specificity values close to 100%, both in non-blind and blind sessions (Table 7). As in experiments using cell lines, the dog correctly identified all tumor and control saliva samples the first time it was exposed to them (Table 5). Finally, Dog#1 demonstrated a similar efficacy in experiments in which the osteosarcoma sample was pooled with 9 negative controls (Table 7).

**Table 7:** Detection of saliva samples from osteosarcoma patients by Dog#1.

		PT#1	PT#2	Saliva pools
All sessions	Sessions	16	8	–
	Trials	168	107	–
	TP	113	64	–
	TN	583	256	–
	FP	3	2	–
	FN	0	1	–
	% Sensitivity (95% CI*)	100 (100–100)	98.46 (95.37–100)	–
	% Specificity (95% CI*)	99.48 (98.67–100.14)	99.22 (98.47–100)	–
Blind sessions	Sessions	6	8	3
	Trials	73	107	36
	TP	53	64	28
	TN	232	256	108
	FP	1	2	0
	FN	0	1	0
	% Sensitivity (95% CI)	100 (100–100)	98.46 (95.37–100)	100 (100–100)
	% Specificity (95% CI)	99.57 (98.97–100.33)	99.22 (98.47–100)	100 (100–100)

*Confidence interval calculated with the values obtained in the different sessions.

## Discussion

In this study we took advantage of the privileged olfactory system of dogs^13^ to detect specific odor signatures with diagnostic potential in sarcomas. Previous studies have already shown the ability of dogs to discriminate cancer-associated VOCs in different types of epithelial cancer^14,15^, however, this the first study demonstrating their potential ability to detect sarcomas. Cancer types with available early screening programs, such as breast, prostate or colon cancer, have significantly improved their survival rates^7–9^. On the other hand, sarcomas are, in general, difficult-to-treat tumors that often develop resistance to current treatments leading to the occurrence of relapses and metastases^2,26,27^. Therefore, the improvement of patient survival for sarcomas largely depends on an early detection at a more curable disease stage. Our study provides a first proof-of-concept that support the development of screening programs for sarcoma based in the detection of specific VOC profiles by sniffer dogs as a reliable non-invasive and costly-effective approach to favor early diagnosis for sarcoma patients.

Our experiments using cell lines, both patient-derived primary cultures and established cell lines, suggest the existence of common odor signatures that can be detected by trained dogs. Several data support the use of culture media from these cell lines as an ideal starting material for training sniffer dogs. First, we and others have demonstrated that these low passaged primary cultures represent close-to-patient models that keep the most relevant genomic and functional alterations of the original tumors^20,28^. Therefore, it could also be expected that most relevant VOCs and odor signatures produced by metabolic processes occurring in tumors are also being produced in cell cultures. Moreover, cell lines, which are exclusively composed by tumor cells, do not contain potential confounding odors produced by other cell types or body fluids substances. Finally, it is well established that MSCs represent the most usual cell of origin for osteosarcomas and other types of sarcomas^3–5^. Therefore, cultures of healthy MSCs represent and ideal choice as non-tumor controls pairs for osteosarcoma cell lines in training and testing experiments. In line with our original hypothesis and our results, a few studies have used before cell lines and/or tumor biopsies from, breast, colon, ovarian or cervical tumors as tumor-only models to train cancer detection dogs with positive results^17,24,25,29^.

The protocols used in this study regarding sample preparation and disposition, dog handling and reward, and data recording were similar to those used in other studies^14,15,17,23,30,31^. After training with an osteosarcoma primary cell line, the sniffer dog was able to detect culture media from other 6 osteosarcoma cells lines not previously used during the training process with sensitivity and specificity rates between 95 and 100%. Moreover, the dog was also able to discriminate saliva samples from two osteosarcoma patients from saliva samples obtained from healthy donors with a similar efficacy. This high specificity and sensitivity rates are in line with the studies showing a more promising ability of sniffer dogs to detect breast, ovarian, prostate or lung cancer^14^.

The results of this study suggest that: (i) there are common odor profiles shared by cultures of osteosarcoma cells obtained from different patients; (ii) VOCs producing these common olfactory signals are circulating throughout the body and can be detected in easily accessible fluids such as saliva; and (iii) these results confirm that the use of media from cell cultures is a useful strategy to train cancer detection dogs. Besides, the experiments performed with positive culture media diluted in control media or with positive saliva samples mixed with healthy samples, revealed that the concentration of cancer specific scents were well above the limit of detection of the canine olfactory system. Moreover, in this study we conducted both, sessions where the trainer was informed about the disposition of the samples and sessions were this information was blinded to the trainer. Our results suggest that in an experimental setting, such as ours, where the dogs are off-leash during the trials, this fact does not have a relevant effect on the results.

Our experimental setup, where multiple consecutive tests were performed with the same positive samples, allowed us to obtain robust data on the sensitivity and specificity of the detection. This repetitive procedure also has associated risks, such as the possibility of a repetitive reinforcement learning of the positive samples by the sniffer dog. However, the fact that our sniffer dogs was able to detect positive samples with very high sensitivity and specificity rates from the first session and that these values remained essentially constant during all sessions performed (Fig. S2), suggest that the reinforced learning associated with the repetition of tests has not played a relevant role in our experiments.

To the best of our knowledge, this is the first study providing a proof of concept about the feasibility of detecting specific cancer scent in saliva. Being able to detect positive sarcoma samples in such an accessible fluid would facilitate the implementation of future screening programs. Osteosarcoma occurs predominantly in adolescents and younger adults between 10 and 19 years^1^. Therefore, screenings could be specifically targeted to school population in this age range. Moreover, we showed that sniffer dogs are able to detect positive saliva samples mixed in a pool of negative samples. If the results of our pilot study are reproduced in future studies analyzing larger number of samples, it can be explored the possibility of speeding the screening process by mixing samples from groups of schoolchildren and subsequently re-analyzing individually only the few mixtures that the dog marks as positive. Beside their possible use in target population screenings, other uses for trained cancer sniffer dogs could be speculated. For instance, scent dogs located in patient’s associations could sniff patients in a regular basis in order to achieve an early detection of relapses in osteosarcoma patients who are in remission.

Although this approach using sniffer dogs could never be considered as a definitive diagnostic method, it could be used as an efficient, rapid and cost-effective pre-screening aid to detect possible cases in a well-defined targeted population and thus contribute to a more efficient use of current diagnostic methods.

Besides, we also hypothesize that combining the olfactory ability of dogs with analytical techniques may lead the way to synergistically improve the detection achieved by each method individually. Thus, the identification of specific compounds or VOC profiles using GC/MS may serve to both: (i) improve dog training to specifically detect these compounds; and (ii) to refine eNose sensors to design novel diagnostic devices applicable to the clinic in the future.

The positive results obtained here must be interpreted taking into account the limitations of the study. An important limitation refers to the fact that only one dog was used in testing experiments. The inclusion criteria led us to select only two dogs. Both of them finished the training process demonstrating high percentages of sensitivity (98 and 91% respectively) and specificity (99 and 85%) in the detection of sarcoma samples. Unfortunately, one of them have to be discarded for testing experiments due to her irregular behavior in training trails. While this is clearly an insufficient sample size, the results obtained with the selected dog provide and initial proof-of-concept about the existence of specific VOC profiles in sarcomas that can be detected by a properly trained dog. In order to establish reliable statistics about the efficiency of our training method and the ability of trained sniffer dogs to detect sarcomas, further research involving a considerable higher number of dogs is needed. Another important limitation of our study is that we used only two saliva samples from osteosarcoma patients to confirm in a clinical setting the results obtained using cell lines. Although, the positive results of this pilot study provides an initial evidence of the feasibility of detecting osteosarcoma-specific odors in saliva samples, conducting new studies that include a large number of saliva samples from healthy controls and patients with osteosarcoma is essential to demonstrate the potential of sniffer dogs to detect osteosarcoma in these types of samples. We have not conducted a sample size calculation in this study; however, our results may be of great use in estimating the number patient samples needed to achieve a significant statistics power in subsequent studies. Finally, to strengthen the results of future studies other distractors, such as samples from other tumor types and/or from patients suffering other bone-related diseases, could be included as controls in training and testing trials.

Overall, our study provides a proof of concept about the existence of a specific cancer scent in osteosarcoma that can be efficiently detected by sniffer dogs trained with osteosarcoma cell lines. Moreover, VOCs producing this odor profile can be detected in easily accessible body fluids such as saliva, which may facilitate the development and implementation of rapid and cost-effective screening methods for the early detection of osteosarcoma.

## Supplementary Information

Supplementary Legends.

Supplementary Figure S1.

Supplementary Figure S2.

## Data Availability

The data presented in this study are contained within the article.
