# Supplementary material for: Proof of concept for the use of trained sniffer dogs to detect osteosarcoma
Source: Sci Rep. 2022 Apr 28;12:6911. doi: 10.1038/s41598-022-11013-1 (PMC9051207; doi:10.1038/s41598-022-11013-1)
Supplement: Supplementary file 1 — Supplementary Legends. [file 41598_2022_11013_MOESM1_ESM.docx]

**Proof of concept for the use trained sniffer dogs to detect osteosarcoma**

**Agustín Ortal el al.**

**LEGENDS TO SUPPLEMENTARY FIGURES**

**Figure S1. Canine scent detection of osteosarcoma cell lines.** Media of the sensitivity (left panels) and specificity (right panels) obtained by the sniffer dog when testing original and diluted OST-3 samples (A) and the panel of osteosarcoma cell lines (B). Error bars represent the standard deviation. One-way ANOVA Turkey’s tests were performed to find statistically significant differences between groups (ns: not significant; *:p < 0.05).

**Figure S2. Percentages of sensitivity and specificity obtained by the sniffer dog in consecutive sessions when testing the indicated osteosarcoma cell lines**.
