# Supplementary figures and images for: Proof of concept for the use of trained sniffer dogs to detect osteosarcoma
Source: Sci Rep. 2022 Apr 28;12:6911. doi: 10.1038/s41598-022-11013-1 (PMC9051207; doi:10.1038/s41598-022-11013-1)

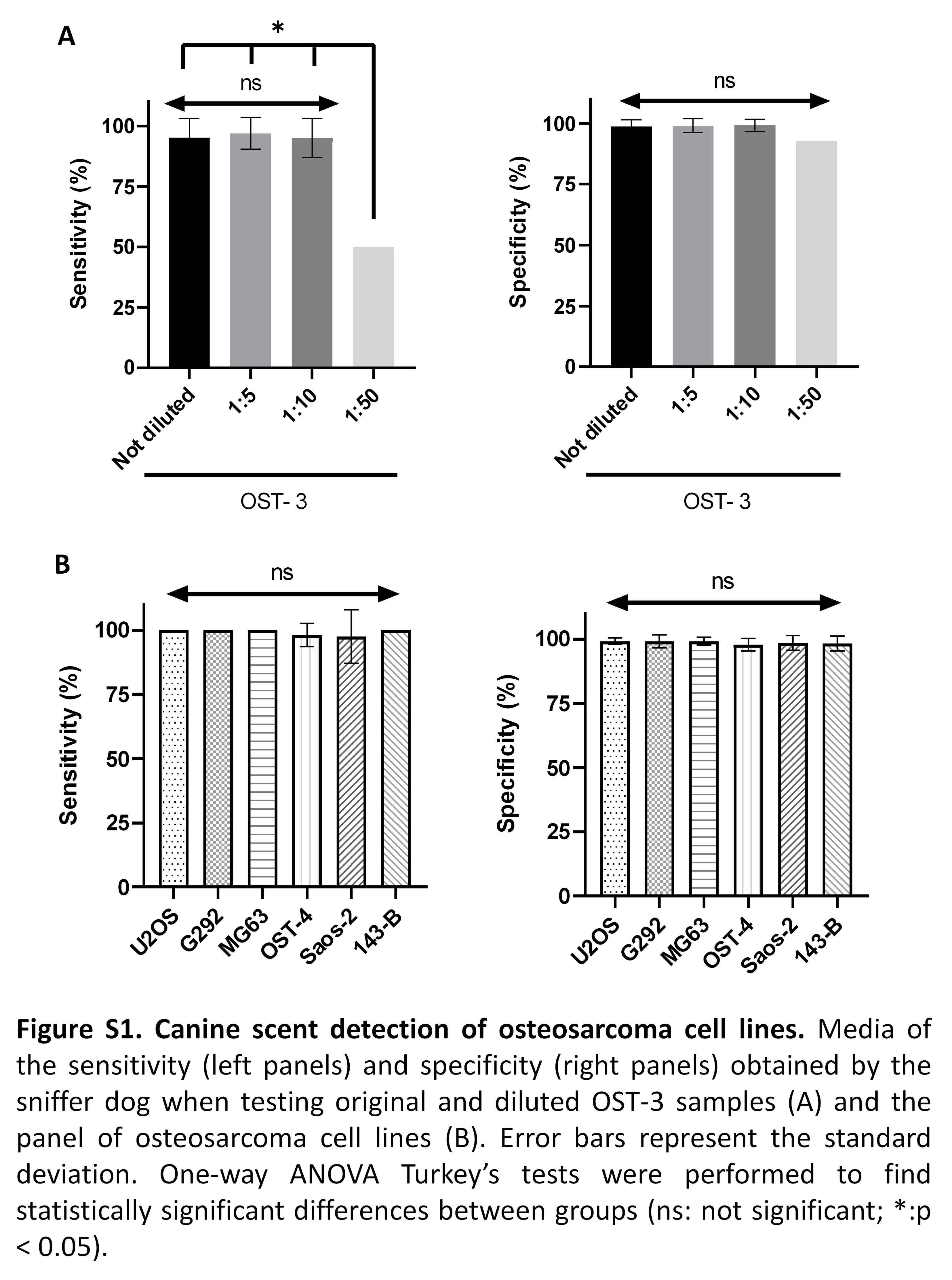

Supplement: Supplementary file 2 — Supplementary Figure S1. [file 41598_2022_11013_MOESM2_ESM.tif]

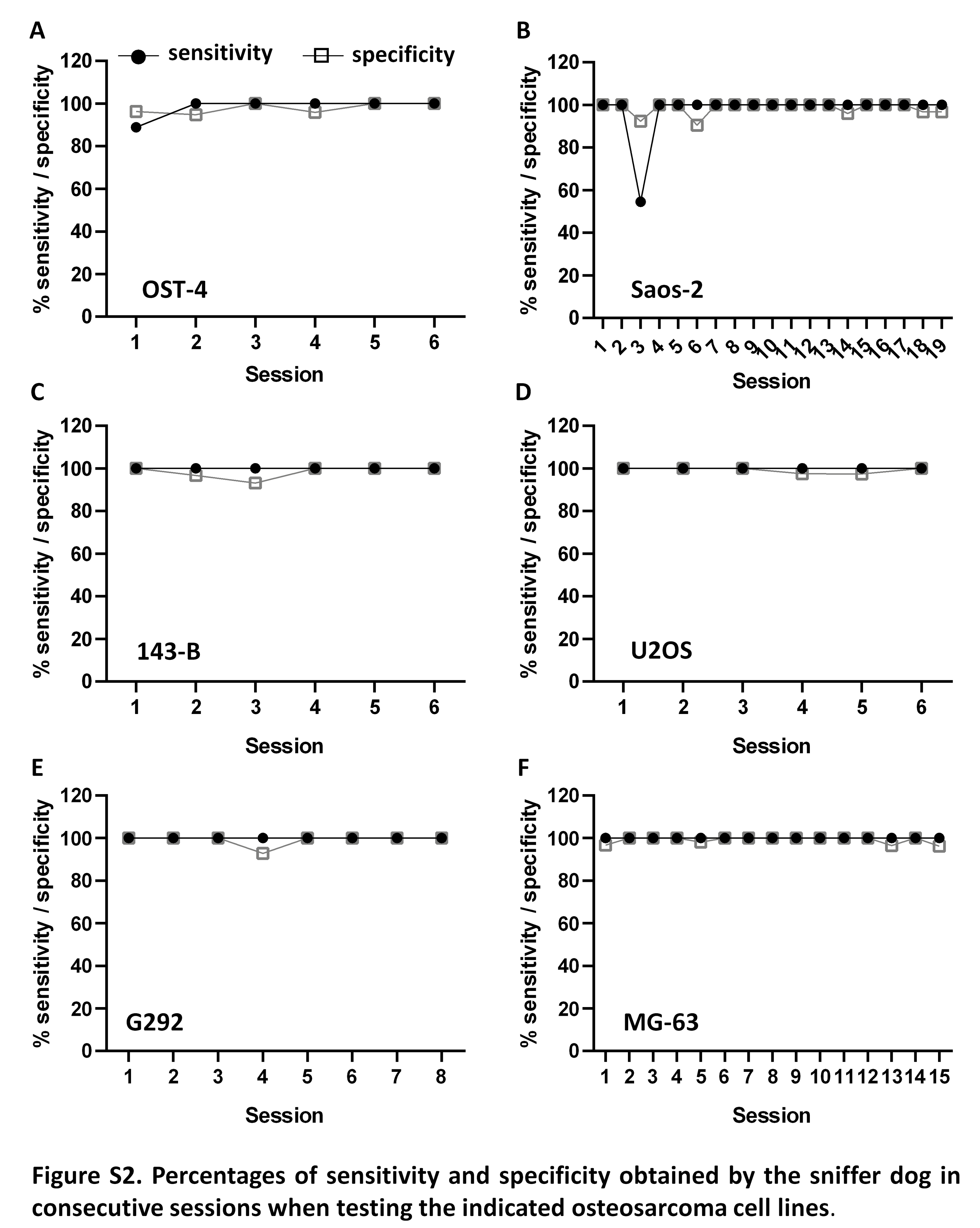

Supplement: Supplementary file 3 — Supplementary Figure S2. [file 41598_2022_11013_MOESM3_ESM.tif]
